# A one-year observational study of all hospitalized and fatal acute poisonings in Oslo: Epidemiology, intention and follow-up

**DOI:** 10.1186/1471-2458-12-858

**Published:** 2012-10-09

**Authors:** Cathrine Lund, Brita Teige, Per Drottning, Birgitte Stiksrud, Tor Olav Rui, Marianne Lyngra, Øivind Ekeberg, Dag Jacobsen, Knut Erik Hovda

**Affiliations:** 1 Department of Acute Medicine, Oslo University Hospital Ullevaal, Kirkeveien 166, Oslo, 0407, Norway; 2 Institute of Forensic Medicine, University of Oslo, Oslo, Norway; 3 Department of Acute Medicine, Lovisenberg Hospital, Lovisenberggata 17, Oslo, 0456, Norway; 4 Department of Medicine, Diakonhjemmet Hospital, Diakonveien 12, Oslo, 0319, Norway; 5 Department of Medicine, Oslo University Hospital Aker, Trondheimsveien 235, Oslo, 0316, Norway; 6 Department of Medicine, Akershus University Hospital, Sykehusveien 27, Nordbyhagen, 1424, Norway; 7 The National Center for NBC Medicine, Department of Acute Medicine, Oslo University Hospital, Kirkeveien 166, Oslo, 0407, Norway

**Keywords:** Epidemiology, Mortality, Paracetamol, Substance abuse, Toxicology

## Abstract

**Background:**

Up to date information on poisoning trends is important. This study reports the epidemiology of all hospitalized acute poisonings in Oslo, including mortality, follow-up referrals, and whether the introduction of over-the-counter sales of paracetamol outside pharmacies had an impact on the frequency of poisonings.

**Methods:**

All acute poisonings of adults (≥16 years) treated at the five hospitals in Oslo from April 2008 to April 2009 were included consecutively in an observational cross-sectional multicentre study. A standardized form was completed by the treating physician, which covered the study aims. All deaths by poisoning in and outside hospitals were registered at the Institute of Forensic Medicine.

**Results:**

There were 1065 hospital admissions of 912 individuals; 460 (50%) were male, and the median age was 36 years. The annual incidence was 2.0 per 1000. The most frequent toxic agents were ethanol (18%), benzodiazepines (15%), paracetamol (11%), and opioids (11%). Physicians classified 46% as possible or definite suicide attempts, 37% as accidental overdoses with substances of abuse (AOSA), and 16% as other accidents. Twenty-four per cent were discharged without any follow-up and the no follow-up odds were highest for AOSA. There were 117 deaths (eight in hospital), of which 75% were males, and the median age was 41 years. Thus, the annual mortality rate was 25 per 100 000 and the in-hospital mortality was 0.8%. Opioids were the most frequent cause of death.

**Conclusions:**

The incidence of hospitalized acute poisonings in Oslo was similar to that in 2003 and there was an equal sex distribution. Compared with a study performed in Oslo in 2003, there has been an increase in poisonings with a suicidal intention. The in-hospital mortality was low and nine out of ten deaths occurred outside hospitals. Opioids were the leading cause of death, so preventive measures should be encouraged among substance abusers. The number of poisonings caused by paracetamol remained unchanged after the introduction of over-the-counter sales outside pharmacies and there were no deaths, so over-the-counter sales may be considered safe.

## Background

Acute poisoning is a common cause of hospital admissions worldwide [1]. The validity of retrospective registry data has been questioned and prospective studies or prospective database registrations are the preferred sources of information [2]. In Oslo, studies of acute poisoning throughout the whole city were performed during 1980 and 2003 [3,4]. The study protocols were similar in both studies where the whole city was monitored over the entire year. Thus, Oslo could provide a reference for such studies and research based on the same protocol is in progress in Yekaterinburg (Russia), Estonia, and Baku (Azerbaijan). The gap between national figures (e.g., the European Monitoring Centre for Drugs and Drug Addiction, EMCDDA) and the picture seen by specialist health care providers is substantial [3,5]. Thus, there is a time lag between the introduction of new substances of abuse and the diagnostics, treatment, prevention, and final legal action required to deal with them.

The in-hospital mortality after a poisoning episode has fallen to 1–2% [4,6], which is most probably due to improved pre-hospital care and hospital intensive care treatment. Therefore, the focus has shifted towards preventing repetition and improving aftercare. One in three patients experiences repeated poisoning within a year and an increased mortality rate has been found even 20 years after a self-poisoning episode [7,8], which emphasizes the importance of adequate follow-up. Furthermore, paracetamol (10 g) was released for around-the-clock over-the-counter (OTC) sales outside of pharmacies in November 2003 (late in the second study). OTC packages accounted for 58% of the total number of doses of paracetamol in 2008 [9]. The relationship between drug availability and suicide attempts is recognized, which made this decision controversial [10]. In 2003, the proportion of paracetamol poisonings in Oslo was 12% (n = 116) [3].

Updated information on poisoning trends and mortality is valuable for clinicians and for establishing preventative initiatives. Most deaths caused by poisoning occur outside hospitals, so including these deaths is important when determining the total mortality. Identifying factors associated with follow-ups may identify targets for improved aftercare. Furthermore, any increase in paracetamol poisonings may result in a re-evaluation of OTC sales.

Our main objectives were as follows: 1) to provide an up to date account of the incidence of hospitalized acute poisonings in Oslo; 2) to document the poisoning pattern and suicide attempts of hospitalized patients, based on age and sex; 3) to study follow-up referrals depending on the reason underlying the poisoning; 4) to calculate the mortality inside and outside hospital; and 5) to determine whether the incidence of paracetamol poisonings increased after the introduction of OTC sales outside pharmacies.

## Methods

### Study design

This study was part of a larger observational cross-sectional multicentre study conducted from April 15^th^ 2008 to April 14^th^ 2009 at the Emergency Medical Agency (EMA, “Oslo Legevakt”, the outpatient emergency clinic in Oslo), the five hospitals that treat poisoned patients in Oslo, and the Institute of Forensic Medicine in the University of Oslo. As Akershus University Hospital is located just outside Oslo and mainly treats patients from outside Oslo, the latter patient group was excluded. This article presents epidemiological data from the hospitals and the Institute of Forensic Medicine. Clinical data derived from the EMA and hospital treatments are presented separately [11,12]. This study had a similar design to another study conducted during 2003. Oslo is the capital of Norway and the population was 568,809 in July 2008, with 466,423 aged ≥16 years. All the hospitals in Oslo belong to the National Public Health Care service. Poisoned patients are not treated in other clinics (e.g., private) than the EMA and the five hospitals.

### Inclusion criteria

All adults (≥16 years) presenting with a primary diagnosis of acute poisoning were included consecutively from the five hospitals. Poisoning was defined as exposure to a substance in toxic amounts. Chronic poisonings were not included. In cases where poisoning was a secondary diagnosis, poisoning itself had to warrant admission. In cases where ethanol and trauma were co-diagnoses, or in doubt, a blood alcohol concentration of 54 mmol/L (250 mg/dL) was used as cut-off. Participation was based on informed verbal consent. Data on deaths occurring in and outside hospitals in Oslo were obtained from the Institute of Forensic Medicine, which is legally obliged to examine all deaths due to poisoning. Permanent residency outside Oslo was not an exclusion criterion in the hospitals located in Oslo. An extensive laboratory screen was performed at the Institute of Forensic Medicine. Drug analyses were performed for hospitalized patients according to the diagnostic needs determined by the treating physician.

### Data collection

Evaluations were conducted by all treating physicians at the five hospitals with the completion of a standardized form that covered the study objectives. Each hospital had a coordinator (MD) who monitored the study and manually cross-checked forms against electronic patient lists to ensure that all patients meeting the criteria were included. In the few cases where patients had been overlooked, a form was completed based on each patient’s electronic medical record. This journal was also used as a supplement when variables were missing. Missing variables were coded as unknown and excluded from that particular analysis.

The data were entered manually into an SPSS spreadsheet and checked systematically. Ten per cent of the variables were cross-checked randomly with the original forms, indicating 99.94% consistency.

### Outcome measures

In cases where patients were exposed to more than one substance, the substance suspected to be present in the most toxic amount was assumed to be the main agent. This was based on the treating physician’s clinical evaluation of all available information, such as statements from patients, companions, outpatient clinics, ambulance services, clinical observations, laboratory findings, or findings at the scene. Substances suspected to be less toxic were registered as co-agents. Carbon monoxide (CO) poisonings were classified as either CO/engine smoke or CO/fire smoke poisonings. Drug screening was performed in all medico-legal autopsies and the main toxic agents were determined by a forensic pathologist. Follow-ups were registered as a categorical variable and transfer to a psychiatric ward was considered the highest level of referral.

The cause of poisoning was evaluated by the treating physician. Poisonings due to substance abuse, such as heroin and ethanol taken for intoxication purposes, were defined as accidental overdoses with substances of abuse (AOSA). Becks’ suicidal intent scale (SIS) was completed if a suicide attempt was suspected. The SIS is a 15-question form used to evaluate the severity of suicidal intention [13]. Each question is scored 0–2, resulting in a final score ranging from 0 to 30. A suicidal intent in poisoning was classified as either a possible or definite suicide attempt. This distinction was based on whether the patient had taken a toxic agent they considered lethal and whether other measures had been taken to ensure a lethal outcome. If the patient reported an ambivalent motive, e.g., by seeking help shortly after ingestion, the motive was considered a possible suicide attempt. Accidents included external causes of poisoning and self-inflicted accidents (e.g., taking the wrong medication) where the agent was not used for self-harm or intoxication purposes. At the Institute of Forensic Medicine, the intention was classified as an open verdict where the intention was unknown. The intention categories differed slightly from the four used in 2003, i.e., definite or possible suicide attempt, appeal for help, and accident/intoxication. Moreover, the SIS was not used in 2003.

### Statistics

Pearson’s chi-square or Fisher’s exact test (cell values <5) were used to compare frequencies. Age comparisons were performed using the Mann–Whitney *U* test.

Univariate logistic regression was used to analyse the association between poisoning intention and the seven major referral end-points: transfer to psychiatric wards, psychiatric outpatient treatment, drug addiction clinics, general practitioners, other follow-up (including transfer to other hospitals, emergency social services, the Norwegian Directorate for Children, Youth and Family Affairs, and other specialists), no referral, and those leaving against medical advice. Subjects who died in hospital or who had unknown intention were excluded from this particular analysis. Findings with *p*-values <0.05 were considered statistically significant. SPSS 16.0 (SPSS Inc., Chicago, IL, USA) was used to analyse the data.

### Ethics

This study was approved by the Norwegian Regional Ethics Committee and the National Data Inspectorate. Studying intoxicated patients is challenging due to the nature of their medical condition, such as the acquisition of written consent. Study subjects were informed about the aims of the study and they were given an information leaflet containing the name and phone number of the study coordinator. They were also given the right to refuse to participate in the study or withdraw consent to participate at any time without reprisal. Their verbal consent was confirmed by the independent interviewing physician.

## Results

Of the 1088 hospitalized patients meeting the inclusion criteria, 23 (2.1%) refused participation. Non-participating patients did not differ significantly from the study population in terms of age, sex, intention, and main toxic agents.

**Figure 1:**
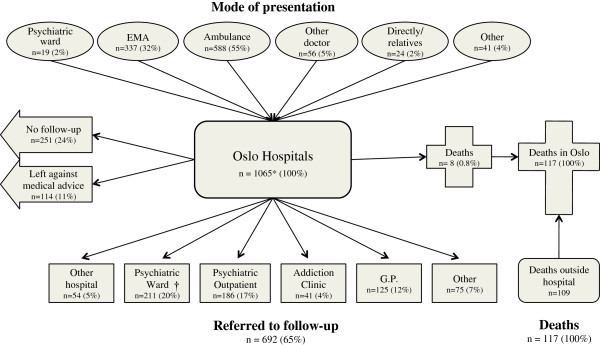
**Patient flow in 1065 hospital admissions for acute poisoning in Oslo in 2008.** *Excludes 23 subjects who refused to participate. †81 transferred under paragraph of compulsory observation. GP: general practitioner; EMA: the Emergency Medical Agency (outpatient clinic). Other presentation includes the police and other institutions. Other referral was mainly the emergency social services.

**Figure 2:**
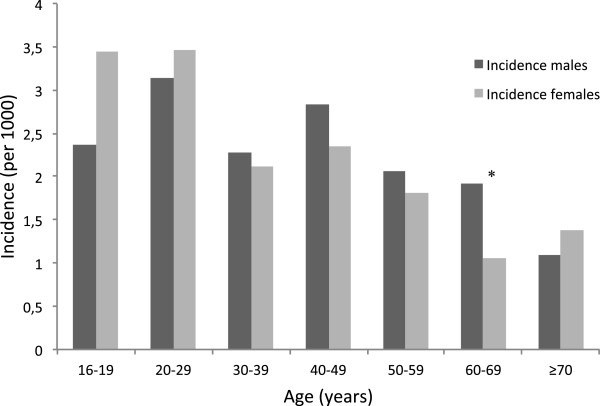
**Incidence according to age and sex in 1065 hospital admissions for acute poisoning in Oslo in 2008.** *Significant difference.

The 1065 hospitalized admissions comprised 912 individuals (Figure 1). Of these admissions, 919 (86%) had permanent residence in Oslo, giving an annual incidence of 2.0 per 1000 inhabitants for both sexes (2.3 per 1000 if all patients were included). The incidence varied in the different age groups, with a peak incidence of 3.1 and 3.5 in males and females aged 20–29, respectively, declining with age (Figure 2). Of the subjects, 460 (50%) were males and the median age was 36 years (range 16–93), i.e., 37 for males and 34 for females (ns). The number of hospital contacts varied, with 822 (90%) being admitted once, 60 (7%) twice, while 30 (3%) were admitted 3–10 times.

The most frequent main toxic agents were ethanol (n = 194, 18%), benzodiazepines (n = 160, 15%), paracetamol (n = 116, 11%) and opioids (n = 114, 11%) (Table 1). Ethanol, opioids, GHB, and amphetamine were significantly more common in males, whereas paracetamol, anti-depressants, and neuroleptics were significantly more common in females. Multiple agents were taken by 593 (56%) and the most common combination was benzodiazepines and ethanol (n = 80, 8%). The most common co-agents were ethanol (n = 251, 24%), benzodiazepines (n = 133, 12%), and benzodiazepine derivates (n = 70, 7%). The toxic agent reflected different intentions. The proportion of possible or definite suicide attempts was highest with paracetamol (n = 101, 87%) and benzodiazepines (n = 102, 64%).

**Table 1:** Total admissions based on agent, and the main agents separated by sex in 1065 hospital admissions for acute poisoning in Oslo in 2008

**Toxic agent**	**Main + co-agents**	**Main agents**			
	**n (%)**	**Total n (%)**	**Males n (%)**	**Females n (%)**	***p*-value**
Ethanol	445 (42)	194 (18)	120 (22)	74 (14)	<0.001*
BZD	293 (28)	160 (15)	73 (14)	87 (16)	ns
Paracetamol^2^	184 (17)	116 (11)	31 (6)	85 (16)	<0.001*
Opioids	152 (14)	114 (11)	80 (15)	34 (6)	<0.001*
GHB	96 (9)	85 (8)	67 (13)	18 (3)	<0.001*
Neuroleptics	99 (9)	60 (6)	15 (3)	45 (9)	<0.001*
Anti-depressants	106 (10)	59 (6)	16 (3)	43 (8)	<0.001*
BZD derivates	128 (12)	58 (5)	24 (4)	34 (6)	ns
CO and fire smoke	50 (5)	48 (5)	31 (6)	17 (3)	ns
Amphetamine	66 (6)	25 (2)	21 (4)	4 (1)	<0.001*
Cardiovascular	27 (3)	18 (2)	8 (1)	10 (2)	ns
Anti-epileptics	48 (5)	17 (2)	5 (1)	12 (2)	ns
Cocaine	30 (3)	13 (1)	10 (2)	3 (1)	ns
NSAIDs	54 (5)	10 (1)	3 (1)	7 (1)	ns
Other gases	13 (1)	11 (1)	4 (1)	7 (1)	ns
Carisoprodol^1^	14 (1)	8 (1)	3 (1)	5 (1)	ns
Scopolamine	9 (1)	8 (1)	4 (1)	4 (1)	ns
Anti-coagulants	8 (1)	7 (1)	3 (1)	4 (1)	ns
Lithium	5 (<0.5)	5 (<0.5)	1 (<0.5)	4 (1)	ns
Antihistamines	54 (5)	4 (<0.5)	0 (0)	4 (1)	ns
Insulin	4 (<0.5)	4 (<0.5)	2 (<0.5)	2 (<0.5)	ns
Other	139 (13)	41 (4)	15 (3)	26 (5)	ns
**Total**		**1065 (100)**	**536 (100)**	**529 (100)**	

*Significant.

^1^Carisoprodol was taken off the market May 1^st^ 2007.

^2^A codeine–paracetamol combination drug was the main agent in 29 cases.

Main agents present in ≤3 admissions were defined as “other”. In six cases, the main agent was unknown.

*BZD* vbenzodiazepines, *GHB* gamma-hydroxybutyric acid, *NSAIDs* non-steroidal anti-inflammatory drugs.

Of the opioids that were main agent, 79 were illegal substances. Of the legal opioids, 14 were methadone and none were buprenorphine. Of the anti-depressants as the main agent, 34 were selective serotonin reuptake inhibitors and 13 were tricycle anti-depressants.

The classification of toxic agent(s) by hospitals was based on statements from patients (n = 759, 71%), laboratory findings (n = 529, 50%), ambulance service or outpatient clinics (n = 387, 36%), companions (n = 275, 26%), clinical observations (n = 205, 19%), or findings at the scene (n = 20, 2%). This variable was missing from seven (1%) forms. The diagnosis was not based on laboratory results alone. However, the diagnosis was based solely on statements from the patient in 203 (19%) cases.

The source of prescription drugs was missing in 349 (30%) cases (Table 2). Paracetamol (non-codeine combination) was purchased OTC in 75 cases (82%) and with a prescription in nine cases (10%). Anti-depressants and neuroleptics were obtained mainly from physicians and more often from general practitioners than psychiatrists. Benzodiazepines were bought illegally in 54 (30%) cases.

**Table 2:** Source of prescription drugs in 801 cases of acute poisoning in Oslo in 2008

	**G.P. n (%)**	**Psych. n (%)**	**Other doc n (%)**	**OTC n (%)**	**Friend/family n (%)**	**Bought illegally n (%)**	**Total n (%)**	**Missing n**
BZD	90 (50)	12 (7)	1 (<0.5)	2 (1)	21 (12)	54 (30)	180 (100)	113
Paracetamol	8 (9)	0 (0)	1 (1)	75 (82)	7 (8)	0 (0)	91 (100)	23
Codeine + paracetamol*	24 (56)	0 (0)	7 (16)	2 (5)	9 (21)	1 (2)	43 (100)	27
Anti-depressants	53 (65)	23 (28)	0 (0)	0 (0)	6 (7)	0 (0)	82 (100)	24
BZD derivates	52 (67)	8 (10)	7 (9)	0 (0)	8 (10)	3 (4)	78 (100)	50
Neuroleptics	41 (55)	31 (42)	1 (1)	0 (0)	0 (0)	1 (1)	74 (100)	25
Legal opioids	16 (39)	0 (0)	1 (2)	0 (0)	4 (10)	20 (49)	41 (100)	16
Anti-epileptics	19 (53)	11 (31)	2 (6)	0 (0)	2 (6)	2 (6)	36 (100)	12
Antihistamines	22 (69)	5 (16)	1 (3)	1 (3)	3(9)	0 (0)	32 (100)	22
Cardiovascular	17 (74)	0 (0)	1 (4)	0 (0)	5 (22)	0 (0)	23 (100)	4
Other	47 (39)	6 (5)	9 (7)	31 (26)	20 (17)	8 (7)	121 (100)	33
Total	389 (49)	96 (12)	31 (4)	111 (14)	85 (11)	89 (11)	801 (100)	349

*BZD* benzodiazepines, *Doc*, doctors, *G.P.*, general practitioner, *OTC* over-the-counter, *Psych*, psychiatrist.

*Combination drug requiring prescription.

No patient reported that they obtained the drug from multiple doctors.

Possible or definite suicide attempts accounted for 495 (46%) admissions (Table 3). The SIS score was used in 349 (71%) of these cases and the median score was 8.0 (interquartile range (IQR) 5–11) for possible and 17.5 (IQR 13–21) for definite suicide attempts. AOSA accounted for 399 (37%) admissions and 170 (16%) were other accidents, of which 99 (58%) were self-inflicted. One patient lacked a recorded intention. There were significantly more records of AOSA in males, whereas possible and definite suicide attempts were more common in females. However, there were no significant sex differences in the SIS scores of subjects with definite or possible suicide attempts. Patients with other accidental poisonings were significantly older than the remainder (difference in the mean 6.2 years, *p* < 0.001). There were no other age differences among the intention categories.

**Table 3:** Physician evaluations of intention in 1065 hospital admissions for acute poisoning in Oslo in 2008

**Intention**	**Males n (%)**	**Females n (%)**	**95 % CI**	**Total n (%)**
AOSA	278 (52)	121 (23)	<0.001*	399 (37)
Possible suicide attempt	115 (21)	229 (43)	<0.001*	344 (32)
Other accidents	87 (16)	83 (16)	ns	170 (16)
Definite suicide attempt	56 (10)	95 (18)	<0.001*	151 (14)
Unknown	0 (0)	1 (<0.5)	ns	1 (<0.5)
Total	536 (100)	529 (100)		1065 (100)

*Significant.

*AOSA* accidental overdoses with substances of abuse, *CI* Confidence interval.

Sixty-five per cent (n = 692) were referred for follow-ups, 24% (n = 251) were discharged without referral, and 11% (n = 114) left against medical advice (Figure 1). Suicide attempt subjects were mainly transferred to psychiatric wards (n = 187, 38%) or referred to psychiatric outpatient clinics (n = 142, 29%) (Table 4). Compared with the suicide attempt subjects, patients with AOSA or other accidental poisonings were more likely to be discharged without follow-up, i.e., OR = 13 (CI 8–20) and OR = 11 (CI 7–18), respectively. Furthermore, patients with AOSA were more likely to leave against medical advice (OR = 4, CI 3–7). Twenty-five (5%) patients with a definite or possible suicidal intention were discharged without any follow-up. None were reported as dead one month after discharge.

**Table 4:** Odds for different follow-ups according to intention in 1002 hospital admissions for acute poisoning in Oslo in 2008. Results of univariate logistic regression

**Intention**	**Psych.ward**	**Psych. OPC**	**Add. clinic**	**G.P.**	**Other**	**No referral**	**Left**
**Suicide attempt** Reference	**38 %**	**29 %**	**4 %**	**9 %**	**10 %**	**5 %†**	5 %
**AOSA** OR (95 % CI)	4 % 0.06* (0.04–0.11)	8 % 0.21* (0.14–0.32)	5 % 1.39 (0.74–2.63)	12 % 1.37 (0.89–2.10)	11 % 1.13 (0.73–1.73)	41 % 13.04* (8.32–20.44)	19 % 4.47* (2.76–7.24)
**Other accidents** OR (95 % CI)	5 % 0.09* (0.05–0.18)	8 % 0.21* (0.11–0.37)	1 % 0.15 (0.02–1.11)	19 % 2.32* (1.42–3.79)	21 % 2.44** (1.52–3.91)	37 % 11.08* (6.66–18.43)	9 % 1.90 (0.97–3.71)
Total	20 %	18 %	4 %	12 %	12 %	24 %	11 %

*Significant, *p* < 0.001, **Significant, *p* < 0.05.

†Two definite suicide attempts.

*AOSA* accidental overdoses with substances of abuse, *CI* confidence interval, *G.P*. general practitioner, Psych. *OPC* psychiatric outpatient clinic.

Both AOSA and other accidents were compared with suicide attempts. The eight patients who died, one patient with unknown intention, and 54 patients transferred to a different hospital were excluded from this analysis.

There were 117 deaths from acute poisoning in Oslo during the study year (Table 5). Of these, 101 (86%) were Oslo residents, giving an annual mortality rate of 25 per 100 000. Eighty–eight (75%) were males and the median age was 41 years (range 18–86), i.e., 41 (range 18–77) for males and 50 (range 19–86) for females (*p* = 0.02).

**Table 5:** Intention and main agents causing deaths from poisoning inside and outside hospitals in Oslo

		**Hospital n (%)**	**Outside n (%)**	**Total n (%)**
Age (years)	Median	42	41	41
	IQR	26–77	29–51	29–51
Sex	Males	2 (25)	86 (79)	88 (75)
	Females	6 (75)	23 (21)	29 (25)
Intention	AOSA	3 (38)	67 (62)	70 (58)
	Other accidents	1 (13)	20 (18)	21 (21)
	Definite suicide	3 (38)	9 (8)	12 (10)
	Possible suicide	1 (13)	9 (8)	10 (9)
	Open verdict	0 (0)	4 (4)	4 (3)
Main agent	Opioids	4 (50)	68 (62)	72 (62)
	CO	1 (13)	10 (9)	11 (9)
	Ethanol	0 (0)	7 (6)	7 (6)
	Codeine (+ paracetamol)*	0 (0)	6 (6)	6 (5)
	Cardiovascular drugs	1 (13)	1 (1)	2 (2)
	Benzodiazepines	1 (13)	3 (3)	4 (3)
	Other illegal	0 (0)	3 (3)	3 (3)
	Anti-depressants	0 (0)	3 (3)	3 (3)
	Anti-epileptics	0 (0)	3 (3)	3 (3)
	Other	1 (13)	5 (5)	6 (5)
Co-agent(s)	Yes	5 (63)	93 (85)	98 (84)
	No	3 (38)	16 (15)	19 (16)
Total		8 (100)	109 (100)	117 (100)

*AOSA* accidental overdoses with substances of abuse, *IQR* interquartile range.

*Combination drug Of the deaths by opioids, nine were due to methadone. The patient who died of benzodiazepines in hospital was 75-years-old, while the three patients who died outside hospital had taken multiple toxic agents. Of the deaths by CO, one was caused by car engine smoke, while the remainder were caused by fire smoke. The death in hospital by “other” was caused by insulin.

Eight patients (0.8%) died during their hospital stay. Six (75%) were females and the median age was 42 years (range 25–86), i.e., 35 for males and 57 for females (ns). The three definite suicides were caused by insulin, CO/fire smoke, and the calcium antagonist amlodipine. The accidental poisoning was caused by benzodiazepines in an elderly patient with dementia.

There were 109 deaths outside hospitals, of which 69 (63%) were found at home, 13 (12%) in a public area, eight (7%) in an institution, and 19 (17%) in other places (e.g., hotel room or a friend’s house). Eighty-six (79%) were males, and the median age was 41 years (range 18–77), i.e., 41 for males and 50 for females (ns). There were significantly more male deaths outside hospital (*p* = 0.002) and the population was younger than those who died in hospital (*p* = 0.026). Sixty-seven (61%) of the deaths were evaluated as AOSA and 49 (73%) of these were caused by opioids.

Paracetamol was considered as the main agent in 116 (11%) hospital admissions, including 29 poisonings with a codeine–paracetamol combination drug, and it was present in 184 (17%) cases (Table 1). No patient with paracetamol poisoning developed liver damage or other sequelae. No patients died of paracetamol poisoning, but six died of poisoning with a codeine–paracetamol combination drug.

## Discussion

### Main summary of results

The incidence of hospitalized acute poisoning in Oslo was 2.0 per 1000, with an equal sex distribution. Almost half of the patients had a suicidal intention. A quarter of the patients were discharged without plans for follow-up, mainly because they were AOSA. The in-hospital mortality was 0.8%. Nine out of ten deaths occurred outside hospitals and opioids were the leading cause of death by poisoning. The number of hospitalized paracetamol poisonings remained unchanged after the introduction of OTC sales outside pharmacies, with no complications or deaths.

### Incidence

Data describing an unselected population of acute poisonings and deaths are sparse, making comparisons difficult. The incidence of hospitalized acute poisonings in Oslo was similar to that in 2003 [3], but marginally lower than the only large prospective study conducted in Europe, which was the Spanish VEIA 2004 study (2.3 per 1000) [14]. In contrast to Spain and most other countries, a separate emergency outpatient clinic (EMA) in Oslo treats the majority of substance abuse poisonings that would otherwise be present in hospital emergency departments (e.g., ethanol, opioid, and stimulants). Twice the number of patients (n = 2348) were treated at the EMA in 2008 compared with all Oslo hospitals, making the total incidence of acute poisonings in Oslo higher [11].

Both sexes were equally represented in our study, which contrasts with the female predominance that is often observed [14] and the male predominance among those treated only in a pre-hospital setting [15].

### Toxic agents

Ethanol and benzodiazepines were the main agents taken, as found in the Spanish VEIA study [14]. Compared with Oslo in 2003, the proportion of opioids (11% vs. 7%, *p* = 0.005) and CO/fire smoke (4% vs. 1%, *p* < 0.001) had increased significantly. However, this observation did not agree with EMCDDA or the United Nations Office on Drugs and Crime (UNODC) reports, which both report estimated declines in the amounts of heroin seizures and opiate use [16,17]. This demonstrates that national figures do not always reflect the local clinical reality. Tricyclic antidepressants (TCA) still contributed to a third of the poisonings by anti-depressants, which agreed with the stable prescription rates over the last five years [9,18]. As often observed, males had more poisonings with substances of abuse, while females had more poisonings with pharmaceuticals such as paracetamol, neuroleptics and antidepressants. However, females did not have more poisonings with benzodiazepines. Surprisingly few patients had taken multiple agents [3].

Half of the prescription drugs were obtained from the patient’s general practitioner, illustrating their potential role in the prevention of acute poisonings. Compared with 1980, more patients had obtained the toxic agent from their general practitioner (49% vs. 36%), whereas fewer were supplied by their family or friends (11% vs. 33%) [19]. Furthermore, none reported that they obtained the agent from multiple doctors, which was the case with 3% of the admissions in 1980. An increased focus on prescription practices, the introduction of a general practitioner scheme in 2001, and the establishment of a central national registry for the prescription of potential drugs of abuse in 2003 have apparently been effective in this respect.

### Suicidal intentions

The observed sex difference in intention was reflected by the toxic agents taken and it may be because males use other more violent and lethal suicide methods to a greater extent [20]. Poisonings with a suicidal intention had increased compared with the 2003 study (14% definite and 32% possible, vs. 10% definite and 25% possible suicide attempts) [3], which was most evident in females. This was supported by statistics from Norway reporting a 23% increase in completed suicides among women from 2003 to 2008, mainly by poisoning, strangulation, and drowning [21]. The same tendency was not seen among men.

### Follow-ups

The proportion of patients referred to follow-ups after a poisoning episode was lower compared with 2003 (75% vs. 82%, *p* < 0.001) [3]. One positive finding was that few patients with a suicidal intention were discharged without follow-up, in accordance with current guidelines. This is important because a previous suicide attempt is the strongest known predictor of completing suicide [22]. However, a quarter of patients who repeatedly poisoned themselves during the 2003-study changed their poisoning intention from suicide attempts to unintentional drug-abuse-related overdoses or vice versa [8], illustrating co-morbidity between substance abuse and suicidal behaviour. The high proportion of substance abusers discharged without follow-up is concerning given the high mortality rate by poisoning and other causes even 20 years after a poisoning episode, irrespective of the intention behind the poisoning [7]. Motivation may be a challenge, but there are follow-up alternatives for overdose victims without suicidal intent in Oslo. Although more follow-ups should be encouraged, the effects on repetition and mortality are incompletely studied.

### Mortality

Both the annual total mortality and the in-hospital mortality rate of acute poisoning were similar to 2003 [23]. A Turkish study reported a similar in-hospital mortality rate of 2.2% [24]. However, as low acuity poisonings were treated at the EMA in Oslo with zero mortality, the in-hospital mortality rate was actually lower [11].

The deaths in and outside hospital were two distinctively different groups. Accidental poisonings of elderly women predominated in hospitals, while AOSA by opiates predominated in middle-aged males outside hospitals. The main cause of death by poisoning in Oslo was opioids taken at home. According to the EMCDDA, Norway is the European country with the most deaths caused by the intravenous use of narcotics per inhabitant [25]. Our finding supports this and highlights a need for prevention initiatives. However, we do not know whether the patients attended their designated follow-up appointments. Moreover, the high proportion of opioid users that left the hospital against medical advice remains a challenge. We will address these aspects in a further study. Pilot naloxone distribution programmes in the US have reported promising results [26], so this may be an important tool for mortality prevention.

### Paracetamol

OTC paracetamol accounted for 82% of the acute poisonings with paracetamol, but only 58% of the total doses sold in Norway in 2008 [9]. This may indicate a correlation between drug availability and self-harm. However, the incidence of hospitalized paracetamol poisonings in Oslo did not change significantly, although the total paracetamol sales in Norway increased by 26% after the introduction of OTC sales outside pharmacies [9,18]. None developed hepatic failure and there were no fatalities. The six deaths linked to paracetamol were caused by a codeine combination drug that required a prescription. Thus, we have no evidence that OTC sales are unsafe when restricted to a package size of 10 g. Our findings support a Canadian study, where the introduction of OTC analgesics in six provinces had no impact on hospital admission [27]. However, paracetamol has been freely available for decades in Australia and studies have reported an increase in overdoses over recent years [28]. Although the case-fatality ratio for paracetamol is low, its widespread use places it among the leading causes of fulminant hepatic failure and drug-related fatalities in both the US and UK [29]. OTC sales are therefore controversial, although there is limited evidence supporting a restriction. In the US, the elimination of prescription paracetamol/opioid combination products is under discussion [30].

### Strengths and limitations

A major strength of this study is that it is prospective, performed within a defined geographical area (capital), and it includes unselected material on all acute poisonings throughout a whole year. Similar studies were performed in the same area during 1980 and 2003, which makes these comparisons more reliable than comparing retrospective studies from different locations.

In terms of limitations, many physicians assisted in the inclusion of patients. This was an advantage when ensuring complete inclusion, but it may have resulted in high interrater variability. A few patients may also have been missed, despite consecutive inclusion, cross-checking of forms against hospital patient lists, and thorough follow-ups.

It is debatable whether a more thorough verification of toxic agents should have been performed. Treatment is mainly symptomatic and not based on the laboratory detection of toxic agents, so this study was based on clinical findings and blood/urine tests used in a routine clinical setting. Furthermore, the limited value of laboratory results in the clinical setting has been demonstrated [31,32]. S-ethanol and s-paracetamol were analysed routinely where they were suspected.

The term “appeal” that was used in the 2003 study was omitted from the present study, because it is both outdated and unspecific. However, this was a limitation in the comparison of suicidal intentions among the hospitalized patients. Furthermore, post-mortem determination of intention is challenging and some suicides might have been classified as accidental deaths and vice versa.

The comparison of paracetamol poisonings with the 2003 study was not optimal because the legislation related to OTC sales was enacted four months before the previous study was completed. However, it took months before the practice was implemented so this comparison may be considered reasonable. Moreover, adolescents <16 years of age were not included in the present study. This may have limited the evaluation of OTC paracetamol sales, as young adolescents are known to have high rates of attempted suicide, potentially with OTC drugs. It was not possible to differentiate between paracetamol bought in and outside pharmacies.

## Conclusions

The annual incidence of hospitalized acute poisonings was 2.0 per 1000 for both sexes, which was not significantly different from 2003. Ethanol and benzodiazepines remained the leading causes of poisoning. The in-hospital mortality remained low and nine out of ten deaths occurred outside hospitals. In total, 46% of the patients had a suicidal intention. Four out of ten patients admitted with an accidental overdose due to substance abuse were discharged without follow-up, which is probably too high given that opioids were the leading cause of death by poisoning. The introduction of OTC sales of paracetamol in packages of 10 g outside pharmacies did not increase the paracetamol-associated poisoning rate or mortality.

## Abbreviations

AOSA: Accidental overdose with substances of abuse; EMA: the Emergency medical agency; EMCDDA: the European monitoring centre for drugs and drug addiction; CI: Confidence interval; CO: Carbon monoxide; IQR: Interquartile range; OR: Odds ratio; OTC: Over-the-counter; SIS: BecksÂ´ suicidal intent scale; TCA: Tricyclic antidepressants; UNODC: United nations office on drugs and crime.

## Competing interests

The authors report that they have no competing interest. The authors alone are responsible for the content and writing of this paper.

## Authors’ contributions

KEH, DJ and OE conceived the study and designed the trial. CL, BT, PD, BS, TOR and ML supervised the conduct of the trial and data collection. CL managed and analyzed the data, including quality control, and drafted the manuscript. All authors read and approved the final manuscript.

## Pre-publication history

The pre-publication history for this paper can be accessed here:

http://www.biomedcentral.com/1471-2458/12/858/prepub
